# Suppressing STAT3 activation impairs bone formation during maxillary expansion and relapse

**DOI:** 10.1590/1678-7757-2023-0009

**Published:** 2023-05-08

**Authors:** Xiaoyue XIAO, Jianwei CHEN, Qiming ZHAI, Liangjing XIN, Xinhui ZHENG, Si WANG, Jinlin SONG

**Affiliations:** 1 Chongqing Medical University College of Stomatology Chongqing Key Laboratory for Oral Diseases and Biomedical Sciences Chongqing China Chongqing Medical University, College of Stomatology, Chongqing Key Laboratory for Oral Diseases and Biomedical Sciences, Municipal Key Laboratory of Oral Biomedical Engineering of Chongqing Higher Education, Chongqing, China.; 2 Sichuan University West China Hospital of Stomatology State Key Laboratory of Oral Disease Chengdu China Sichuan University, West China Hospital of Stomatology, State Key Laboratory of Oral Disease and National Clinical Research Center for Oral Diseases, Chengdu, China.

**Keywords:** Bone formation, Maxillary expansion, STAT3 protein

## Abstract

**Objectives:**

The mid-palatal expansion technique is commonly used to correct maxillary constriction in dental clinics. However, there is a tendency for it to relapse, and the key molecules responsible for modulating bone formation remain elusive. Thus, this study aimed to investigate whether signal transducer and activator of transcription 3 (STAT3) activation contributes to osteoblast-mediated bone formation during palatal expansion and relapse.

**Methodology:**

In total, 30 male Wistar rats were randomly allocated into Ctrl (control), E (expansion only), and E+Stattic (expansion plus STAT3-inhibitor, Stattic) groups. Micro-computed tomography, micromorphology staining, and immunohistochemistry of the mid-palatal suture were performed on days 7 and 14. *In vitro* cyclic tensile stress (10% magnitude, 0.5 Hz frequency, and 24 h duration) was applied to rat primary osteoblasts and Stattic was administered for STAT3 inhibition. The role of STAT3 in mechanical loading-induced osteoblasts was confirmed by alkaline phosphatase (ALP), alizarin red staining, and western blots.

**Results:**

The E group showed greater arch width than the E+Stattic group after expansion. The differences between the two groups remained significant after relapse. We found active bone formation in the E group with increased expression of ALP, COL-I, and Runx2, although the expression of osteogenesis-related factors was downregulated in the E+stattic group. After STAT3 inhibition, expansive force-induced bone resorption was attenuated, as TRAP staining demonstrated. Furthermore, the administration of Stattic *in vitro* partially suppressed tensile stress-enhanced osteogenic markers in osteoblasts.

**Conclusions:**

STAT3 inactivation reduced osteoblast-mediated bone formation during palatal expansion and post-expansion relapse, thus it may be a potential therapeutic target to treat force-induced bone formation.

## Introduction

Insufficient maxilla width often leads to posterior crossbite and/or dental crowding. Treatment modalities, such as rapid maxillary expansion (RME), were implemented for correction. RME appliances subject the mid-palatal suture (MPS) to heavy forces, separating it and achieving new bone formation within this region. To ensure sufficient time for bone formation and maintain the stability of RME, a prolonged retention period, in which appliances are kept *in situ* for up to six to eight months is routinely conducted. However, poor patient cooperation, impaired oral hygiene, and appliance breakage due to prolonged retention are common in clinics, which could compromise RME treatment outcomes.

Mechanical stimuli imposed on MPS initiate bone remodeling accompanied by fiber and cartilage rearrangements. Under general conditions, force-induced bone remodeling continues until bone formation and resorption reach equilibrium. To address this relapse issue, previous studies mainly focused on investigating supplementary methods, such as growth factors, drugs, and low-level laser therapy, to accelerate bone formation following RME in animal models.^1,2^ However, information on the conversion of mechanical stimulation into biochemical signals during this process is scarce. The identification of mechanosensory molecules could help develop mechanism-based adjuvant methods to promote bone formation. Thus, it is essential to understand the cellular and molecular events during RME and post-expansion relapse.

Signal transducer and activator of transcription 3 (STAT3) is implicated in bone growth and metabolism. As a nuclear transcription factor ubiquitously expressed in bone tissues, the activation of STAT3 promotes osteoblast differentiation and bone defect healing.^3,4^ STAT3 mutations impair bone development, decreasing bone mineral density and recurrent bone fracture.^5^ STAT3 has mechanical properties. For example, mechanical stretch-induced activation of STAT3 was observed in cardiomyocytes.^6^ STAT3 is expressed in osteocytes and mediates force-induced bone formation in long bone^7^ and orthodontic tooth movement.^8^ Moreover, it is unclear so far whether STAT3 participates in RME-induced MPS and, if so, what exactly performs in bone formation.

Therefore, we first clarified tissue reconstruction in MPS during RME and post-expansion relapse by establishing a rat model. The effect of Stattic (a STAT3-specific inhibitor) on bone formation was further analyzed to elucidate the role of STAT3 *in vivo*. Since osteoblast-mediated bone formation is imperative in bone remodeling, we exposed rat primary osteoblasts to mechanical stretching and examined the involvement of STAT3.

## Methodology

### Animal specimens

The study sample consisted of 30 male Wistar rats aged five weeks, with an average weight of 100±10 g. The animals were randomly allocated to three groups (n=10): Ctrl (control; no expansion appliance), E (expansion only), and E+Stattic (expansion + STAT3 inhibition) groups. The experimental protocol was approved by the Animal Ethics Committee of West China Hospital of Stomatology of Sichuan University (WCHSIRB-D-2021-193).

**Figure 1:**
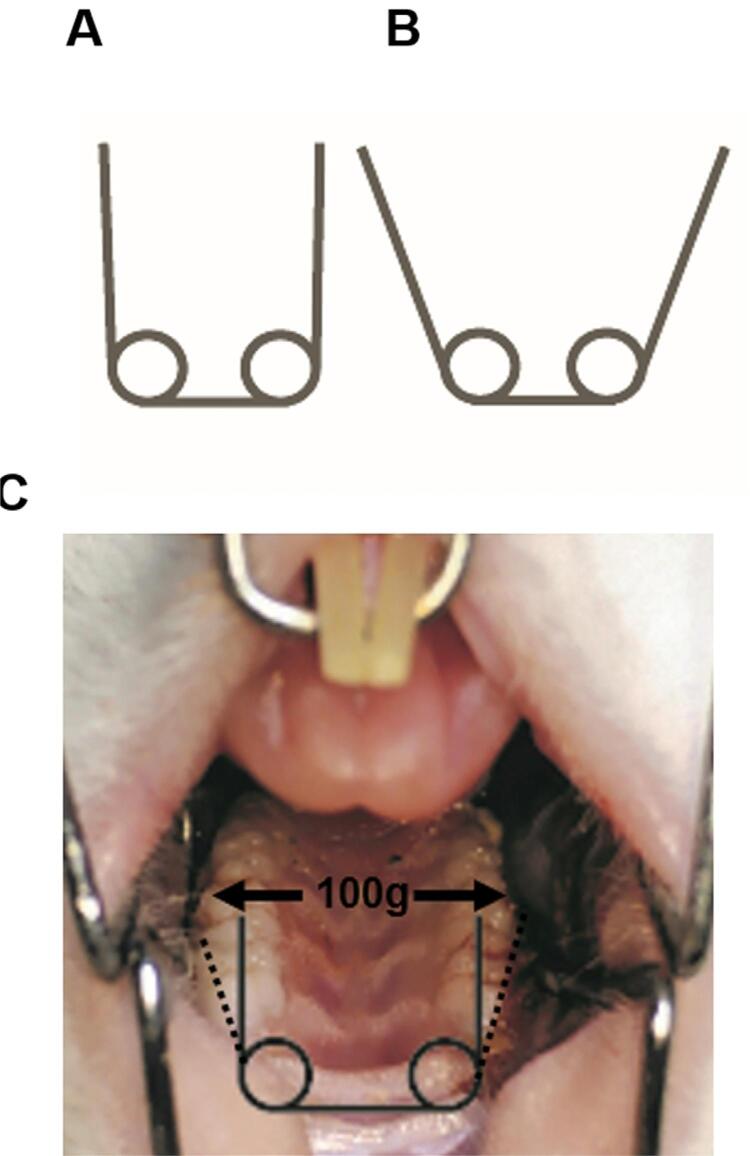
Schematic image of expansion appliances used in the study. A, Inactivated expander. B, Activated expander. C, 100 g expansion force was exerted when the expander was installed on the occlusal surface of molars

A customized expansion device was fabricated as described previously^9,10^ by bending a 0.014-inch Australian wire into a two-eye open spring. When the expander was inactivated, both arms were parallel (Figure 1A). To activate the expander, the two arms were pulled aside symmetrically (Figure 1B). Under anesthesia with a combination of ketamine (87 mg/kg) and xylazine (13 mg/kg), the expander was bonded onto the occlusal surfaces of rat molars with its two arms in parallel and exerting a force of 100±5 g (Figure 1C). After 7-day expansion, the expanders were detached, and the rats experienced another 7-day relapse. Five rats from each group were euthanized on days seven (D7) 7 and 14 (D14).

### Inhibitor solution and *in vivo* administration

Stattic (Selleck, USA) was dissolved in dimethyl sulfoxide, PEG 300, Tween 80, and ddH_2_O according to the manufacturer’s instructions. Then, 10 μL Stattic solution was locally injected beneath the mucoperiosteum in the middle of the MPS between the first molars. From day one (D1), the E+Stattic group received daily injections, and rats in the Ctrl and E groups were given an equivalent volume of the placebo vehicle.

### Arch width measurement

The arch width was defined as the distance between the gingival margins of the first molars. On D0, D7, and D14, the arch width of each rat was measured using a vernier caliper.

### Specimen collection

Randomly selected rats from each group were euthanized at D7 and D14. Maxillae were anatomized and fixed with 4% paraformaldehyde for 48 h at 4°C. After fixation, the samples were subjected to micro-computed tomography (Micro-CT) scanning. After Micro-CT scanning, the samples were decalcified in 10% EDTA for approximately two months. Dehydration and paraffin embedding were performed using conventional techniques. Sagittal serial sections of the MPS (3-μm thick) were obtained within the first molar region.

### Micro-computed tomography scanning

Micro-CT scanning was performed with the system (Scanco Medical, Wangen-Brüttisellen, Switzerland, scanning voltage: 90 kV, current: 50 μA, resolution: 10 μm). The region of interest (ROI) began from the mid-palatal bony edges bilaterally extending 200 μm on each side, and was within the first molar root region in the anterior-posterior direction. The bone volume/tissue volume ratio (BV/TV) and trabecular number (TbN) were estimated using innate software (VG Studio Max Software, Heidelberg, Germany) with a threshold of 212-1000. The same settings and thresholds were applied to every sample to ensure consistency.

### Histomorphology staining

After dewaxing and rinsing with water, paraffin slices of the maxillae were subjected to HE staining (Solarbio, China). A Masson’s trichrome staining kit (Solarbio, China) was used to visualize fibrous tissues in the MPS. All procedures were conducted according to the manufacturer’s instructions. The sections were treated with Ponceau-Acid Fucshin solution for five minutes, followed by differentiation in phosphomolybic acid solution until collagen was not red. Sections were then soaked in aniline blue solution and then washed with acetic acid working solution. The cartilage reconstruction in MPS was illuminated using a modified Russell-Movat pentachrome stain kit (Abcam, USA). The instructions were strictly followed and every procedure was checked under a microscope. Images of MPS within the edges of the palatal bones, as well as the periosteum on the oral and nasal side, were captured with the ECLIPSEE200 microscope (Nikon Instruments, Melville, NY, USA).

### TRAP staining

TRAP staining of the sections was carried out using the TRAP/ALP staining kit (Fujifilm WAKO, Japan) according to the manufacturer’s instructions. The number of TRAP-positive cells was estimated and expressed as cell numbers per millimeter in the MPS (Image-pro Plus, Media Cybernetics, USA).

### Immunohistochemical staining

Immunohistochemical staining of COL-I, Runx2, and ALP was conducted as previously described.^11^ The sections were treated with 3% H_2_O_2_ for 30 min at room temperature in the dark and washed three times with PBS. After 30 min incubation at room temperature with block solution containing 4% bovine serum albumin, sections were then soaked with primary antibodies dissolved in blocking solution: COL-I (dilution 1:150; ET1609-68), Runx2 (dilution 1:400; ET1612-47), and ALP (dilution 1:400, ET1607-53) from HuaBio (Hangzhou, China) at 4°C overnight. On the next day, the slides were incubated with secondary antibodies at room temperature for 50 min then rinsed with 3,3’-diaminobenzidine (DAB). Negative controls were used to validate the specificity of the immunoreactions. The means of integrated optical density (IOD) were quantified using Image-Pro Plus 6.0 (Media Cybernetics, Bethesda, MD, USA).

### Cell culture and treatment

Rat primary osteoblasts were obtained from the calvarial bones of neonatal Wistar rats.^12^ Calvarial bones were washed with PBS supplemented with 100 U/mL penicillin and 100 μg/mL streptomycin at least twice. Then, the bones were incubated with 0.25% trypsin at 37°C for 30 min, dissected and digested with 0.1% collagenase I at 37°C overnight. The cell suspension was harvested and osteoblasts were cultured in complete medium consisting of α-MEM, 10% FBS, 100 U/mL penicillin, and 100 μg/mL streptomycin.

For *in vitro* mechanical stretch application, cells were seeded onto collagen I-coated six-well BioFlex plates (Flexcell Int. Corp. Hillsborough, NC, USA). The loading regime was 10% magnitude, 0.5 Hz frequency, and 24 h duration. The osteoblasts were divided into Ctrl, cyclic tensile stress (CTS), and CTS+Stattic groups. For STAT3 inhibition, 5 μM Stattic was added to the test medium.

### Western blotting

Proteins were extracted from the osteoblasts of each test group. The procedure was conducted according to a previous study.^13^ The primary antibodies used were anti-ALP (1:1000 dilution, ET1607-53), anti-Runx2 (1:2000 dilution, ET1612-47), anti-Osterix (1:1000 dilution, ER1914-47), anti-COL-I (1:1000 dilution, ET1609-68), anti-STAT3 (1:4000 dilution, ET1607-38), anti-pSTAT3 (1:4000 dilution, ET1603-40), anti-JAK2 (1:1000 dilution, ET1607-35), anti-pJAK2 (1:1000 dilution, ET1607-34), and anti-GAPDH (1:5000 dilution, ET1601-4). Protein samples were separated using sodium dodecyl sulfate-polyacrylamide gel electrophoresis and transferred to polyvinylidene fluoride membranes. Membranes were blocked with fat-free milk for 1 h and then incubated with primary antibodies at 4°C overnight. On the next day, blots were incubated with horseradish peroxidase-conjugated secondary antibodies (1:1000 dilution) for 1 h and detected using enhanced chemiluminescence.

### Alkaline phosphatase and alizarin red staining

Osteoblasts in all groups were cultured in mineralization media and harvested to detect the mineralization capacity. An ALP assay kit (Beyotime, Shanghai, China) was used according to the manufacturer’s instructions and alizarin red staining performed as previously described.^11^ The cells were rinsed with PBS thrice and fixed with 4% paraformaldehyde, followed by alizarin red staining (Sigma-Aldrich, St. Louis, USA). Images were visualized under a light microscope (Olympus IX71, Tokyo, Japan).

According to the previously published study,^11^ Image J was used to analyze ALP activity and alizarin red staining images quantitatively. Images were converted into “RGB stack” and the threshold was adjusted to identify mineralization areas. The ratio of mineralization areas was our readout.

### Statistical analysis

Data were expressed as the mean ± standard deviation collected from three independent experiments. Inter-group comparisons were performed using Student’s t-test (two groups) and one-way analysis of variance (three or more groups). Statistical significance was set at P<0.05 (version 7, GraphPad Software, Inc., La Jolla, CA, USA).

## Results

### STAT3 inactivation negatively affected the efficacy of palatal expansion and mid-palatal suture bone microstructure

**Figure 2:**
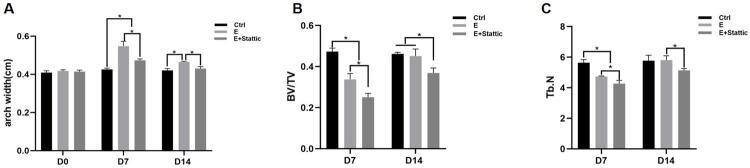
Arch width changes and Micro-CT analysis. A, Arch width measurements. B, BV/TV change. C, TbN value change. *P<0.05

On D0, the baseline arch widths in all groups were consistent. After 1-week expansion, the E and E+Stattic groups showed increased arch width compared to that of the Ctrl group. The arch width of the E group was significantly greater than that of the E+Stattic group (p<0.05). For the E and E+Stattic groups, the arch width decreased after 1-week relapse. However, the arch width of the E group at D14 was significantly greater than that of the other two groups (Figure 2A, p<0.05).

Regarding BV/TV and TbN, the E and E+Stattic groups showed decreased measurements on D7. After 1-week relapse, both measurements for the E group returned to those of the Ctrl group, whereas the two experimental groups showed significant differences (Figure 2B, C; p<0.05).

### Inhibition of STAT3 impacted tissue remodeling in the mid-palatal suture evoked by palatal expansion

**Figure 3:**
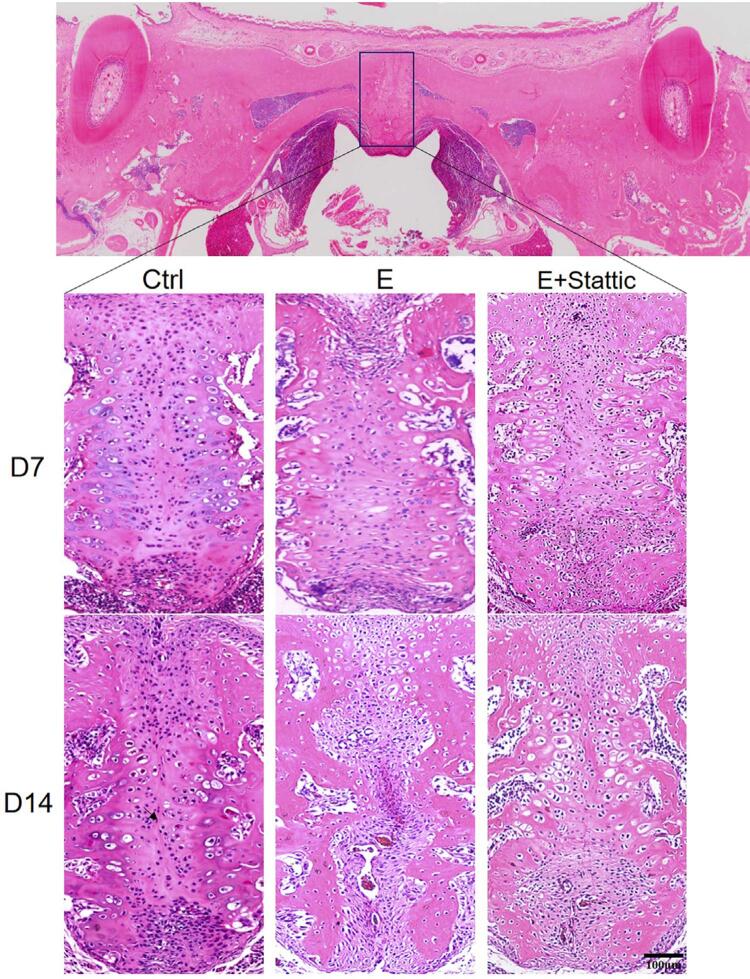
HE staining of the mid-palatal suture

First, HE staining was performed to examine morphological changes in the MPS. The MPS structure of the Ctrl group consisted of cartilage on both sides and a thin layer of fibrous tissue on D7 and D14. The periosteal cells on the oral and nasal sides were distinct. In the E group, chondrocytes were separated and periosteal cells migrated to the MPS after 1-week expansion. On D14, the width of the suture decreased as the periosteal cells continued to increase and accumulate in the MPS. In the E+Stattic group, periosteal cell migration and accumulation was lower on D7 compared to that of the E group. Furthermore, reorientation parallel to the mechanical force was less distinguishable. After 1-week relapse, periosteal cells were mainly located near the nasal side. The suture in the MPS returned to its original state, comprising layers of chondrocytes separated by narrow fibrous tissues (Figure 3).

**Figure 4:**
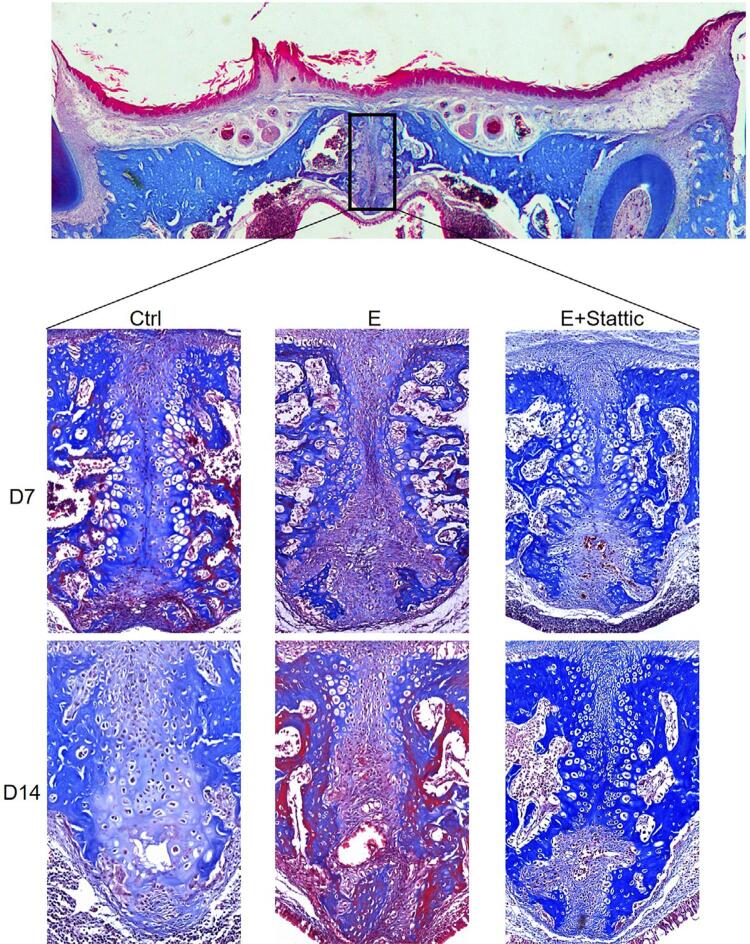
Masson staining of the mid-palatal suture

Masson’s trichrome staining presented fibrous tissue reconstruction within the MPS. In the Ctrl group, a thin layer of fibrous tissue was found in the middle of the MPS. Minor changes were observed between D7 and D14. After 1-week expansion, the fibrous tissue in the E group was reoriented and expanded parallel to the mechanical force. However, the reorientation trend decreased after relapse on D14. In the E+Stattic group, the reorientation induced by mechanical loading was disturbed on D7 and D14 (Figure 4).

**Figure 5:**
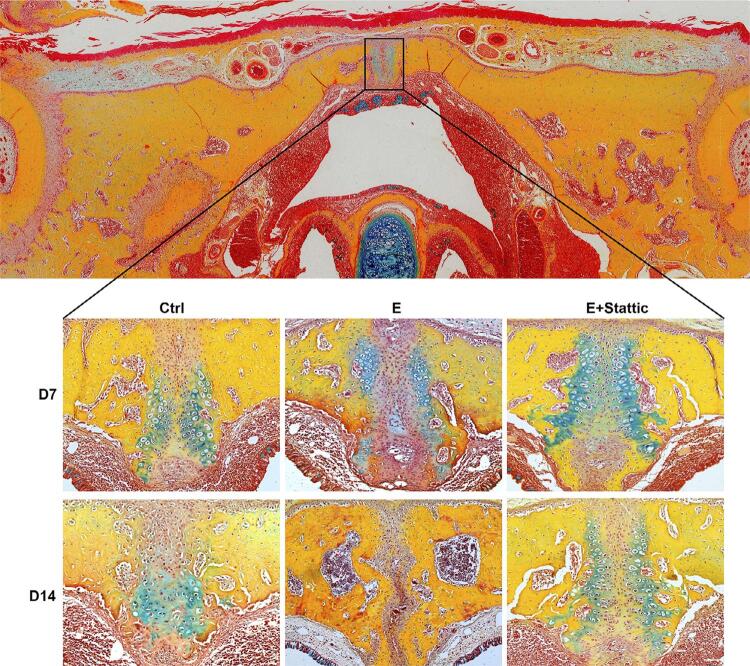
Russell-Movat pentachrome staining of the mid-palatal suture

Figure 5 showed that chondrocytes were stained bright blue after Russell-Movat pentachrome staining. As the rats grew, the two lateral cartilage masses and fibrous tissue in the Ctrl group became more interlinked from D7-D14. Chondrocytes in the E group were forced apart laterally after 1-week expansion and their number decreased compared to that of the Ctrl group. Notably, chondrocytes number continued to decrease and were almost indiscernible after relapse. Compared with that of the E group, the decrease in chondrocyte number in the E+Stattic group was less noticeable after expansion. Corroborating the findings of HE staining, reoccurrence of bilateral chondrocytes was observed in the E+Stattic group after relapse (Figure 5).

### Enhanced bone remodeling induced by palatal expansion was attenuated by STAT3 inhibition

**Figure 6:**
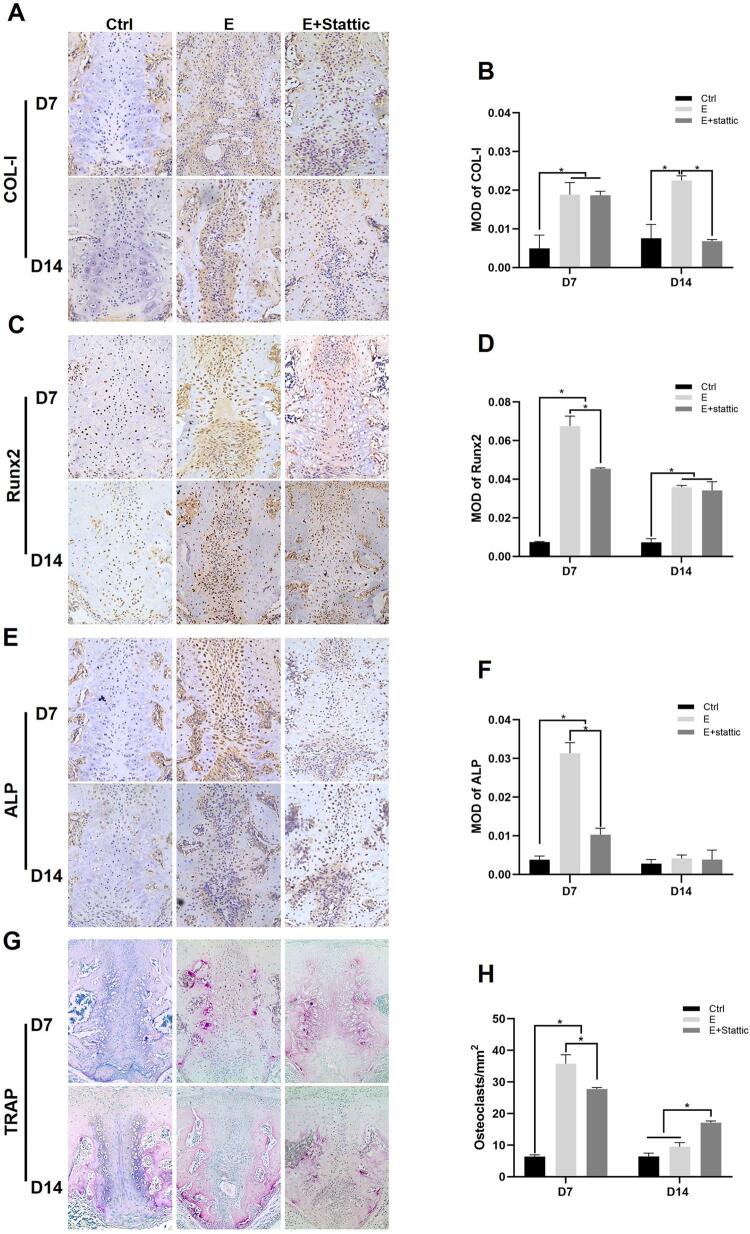
Palatal expansion and STAT3 inhibition influence osteogenesis related factors and osteoclast activity. A, C, E, Immunohistochemical staining of COL-I, Runx2, and ALP. B, D, F, Semiquantitative analysis of COL-I, Runx2, and ALP expression in the mid-palatal suture. G-H, Representative images of TRAP staining and TRAP-positive cells count. *P<0.05

The intensity of osteogenic markers (ALP, Runx2, and COL-I) was examined using immunohistochemical staining. After 1-week expansion, the expression levels of ALP, Runx2, and COL-I were all upregulated. The inhibition of STAT3 partially reversed the increased expression of Runx2 and ALP, but their staining intensities were still higher than those of the Ctrl group. On D14, the expression of COL-I and Runx2 in the E group was greater than that in the Ctrl group. No significant difference was found in the ALP staining intensity between each group on D14 (Figure 6A-F).

TRAP staining was used in this study to demonstrate osteoclast activity. No obvious bone resorption was observed on D7 and D14 in the Ctrl group (Figure 6G). In the E and E+Stattic groups, TRAP-positive cells were mainly observed on the outer side of hypertrophic chondrocytes and along the marrow cavity. After 1-week expansion, TRAP-positive cells increased significantly, but then returned to the Ctrl level on D14. The E+Stattic group displayed fewer TRAP-positive cells than the E group on D7, whereas on D14, the number of TRAP-positive cells in the E+Stattic group was significantly higher than the E and Ctrl groups (Figure 6H).

### Cyclic tensile stress-responsive calvarial osteoblasts showed higher expression of osteogenesis-related markers and mineralization capacity, and were attenuated by STAT3 inhibition

**Figure 7:**
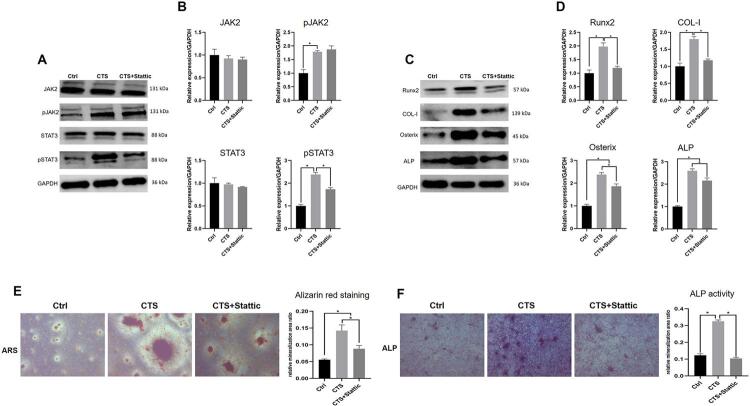
Osteoblastic STAT3 mediated the mechanical loading-increased osteogenesis. A-B, The protein levels of JAK2, pJAK2, STAT3, and pSTAT3. C-D, The protein levels of Runx2, COL-I, Osterix, and ALP. E-F, Alizarin red staining and ALP staining. *P<0.05

First, the patterns of JAK2/STAT3 protein expression were assessed using western blotting. STAT3 protein expression was similar in all groups, whereas pSTAT3 expression increased after mechanical loading. Moreover, JAK2, an upstream molecule of STAT3, was also phosphorylated by mechanical stimuli (Figure 7A, B). Then, osteogenic molecule expression and mineralization capacity were examined. Tensile stress upregulated the expression of Runx2, COL-I, Osterix, and ALP, which was partially reversed by STAT3 inactivation (Figure 7C, D). The CTS group also exhibited increased ALP staining intensity and more mineralized nodules than the Ctrl group. However, Stattic administration decreased this promoting effect (Figure 7E, F). These results confirmed that STAT3 modulated osteogenesis was enhanced by tensile stress.

## Discussion

RME has been widely implemented by orthodontists for maxillary transverse deficiency in growing individuals. However, the high relapse rate due to insufficient bone formation/mineralization remains a prime concern. To minimize relapse, various interventions have been used in animal models to augment bone formation, such as treatment with parathyroid hormone,^14^ *Salvia officinalis* L. extracts,^15^ and salvianolic acid B.^16^ These biological factors and drugs should stimulate bone formation in MPS subjected to RME. Thus, it is crucial to investigate the intrinsic mechanisms that drive bone formation. In the present study we identified that STAT3 participates in RME-induced bone formation by playing an anabolic role in osteoblasts, which could shed light on basic biological signaling events and aid in developing more advanced and precise pro-osteogenic factors.

Corroborating previous findings,^17^ the control suture consisted of cartilage-covered palatine connected by a thin band of fibrous tissue. In our study, 1-week *in-situ* expansion increased osteoblast-like cell proliferation, indicating active bone formation. Osteoblast-like cells, referred to as periosteal cells,^18^ were the major mechano-responsive functional cells in the context of RME. Cell fate mapping studies have revealed that periosteal cells generate chondrocytes and osteoblasts during bone regeneration, depending on the local environment.^19,20^ Histomorphological changes during expansion and post-expansion relapse in our study were justifiable. Following RME, periosteal cells migrated to MPS, proliferated, and differentiated into bone-forming osteoblasts. Relapse often occurs due to compressive forces from adjacent structures, such as circummaxillary sutures and buccal muscule.^21^ The MPS after relapse showed a densely aggregated cell population, probably due to its tendency to return to the previous suture width. These observations correspond with the histological changes reported in other studies.^22^ However, STAT3 inhibition disrupted these normal histological changes. Periosteal progenitors are committed to the osteogenic lineage with upregulated STAT3 phosphorylation.^23^ STAT3 hyperactivation accentuates bone formation on the periosteal surface in response to mechanical stimuli.^24^ During both the expansion and relapse periods of our study, STAT3 inactivation reduced migration of periosteal cells to the MPS and suppressed suture widening. Thus, the close relationship between STAT3 and periosteal cells may also impact bone formation in the MPS.

Secondary cartilage has been a topic of interest for decades because its biomechanical properties are the bedrock of many orthopedic and orthodontic techniques.^25^ The MPS, located between the bilateral maxillary bone, contains secondary cartilage, and its response to forces remains incoherent in the literature. Chondrocytes in non-expansive MPS follow a certain distribution pattern: progression from small-sized chondrocytes near the middle to hypertrophic chondrocytes close to the bony edge. Some studies found that expansive forces induced cartilage degeneration and reduced proteoglycan content, suggesting that the cartilage was absorbed and then replaced by bone.^17,26^ Other studies have reported that the cartilage range is increased and chondrocytes are more proliferative under expansive forces.^27,28^ Both endochondral and intramembranous ossifications participate in bone remodeling processes in the MPS.^29^ In cases of endochondral ossification, hypertrophic chondrocytes undergo absorption, invasion of new tissues, and mineralization. In this study, we found newly formed bone matrix between the cartilage and maxilla bone to replace hypertrophic chondrocytes after 1-week expansion. The STAT3 pathway positively regulates chondrogenic differentiation^30^ and its loss of function damages normal endochondral ossification in the skeletal system.^31^ Thus, the unchanged number of hypertrophic chondrocytes in this study after STAT3 inhibition might imply impeded endochondral ossification in the MPS. After appliance removal, we found immature chondrocytes with a high nucleus-to-cytoplasm ratio along with a concentrated osteoblast population. These chondrocytes can be proliferative and hypertrophic.^32^ After expansion, they secrete abundant extracellular matrix, which restores cartilage structure.

Physiologically, the periosteal cells and pre-existing mesenchymal cells in the precartilage layer could contribute to bone formation under expansive forces.^33^ Several distinct subpopulations of suture mesenchymal cells have been identified and they have subtly different biological properties.^34^ There is limited availability for the isolation and culture of suture mesenchymal cells from the MPS region. Moreover, heterogeneity between mesenchymal cell subsets is probably a confounding variable. Osteoblasts are the major bone-forming cells and differentiated from mesenchymal cells. Thus, to minimize confounding factors, we examined the mechanisms behind mechanical loading, STAT3, and their roles in bone formation using rat primary osteoblasts. STAT3 deletion decreases bone mineral density^5^ and slows orthodontic tooth movement by impeding osteoblast/osteoclast function in alveolar bone remodeling.^8^ In this study, osteoblasts were force-loaded with the Flexcell Tension system to mimic the expansion force *in vitro* and screened for mechanosensitive characteristics of the STAT3 pathway. Upon mechanical stimulation, pJAK2 and pSTAT3 showed increased expression, as evidenced by western blotting. The activation of JAK2/STAT3 in response to mechanical loading is also present in periodontal ligament fibroblasts^35^ and cardiomyocytes.^6^ However, evidence about how mechanical loading activates JAK2/STAT3 is scarce. In cardiomyocytes, loading-induced JAK2/STAT3 activation is correlated with the interleukin-6 (IL-6) family, autocrine/paracrine-secreted angiotensin II, intracellular Ca^2^ level, and protein kinase C.^6^ Liu, et al.^36^(2017) highlighted the critical role of IL-6 in STAT3 phosphorylation in orthodontic tooth movement. Thus, information on the precise mechanism by which mechanical loading induces phosphorylation of JAK2/STAT3 is scarce. Further investigations are necessary to clarify this.

Pharmacological inhibition of STAT3 directly undermined osteoblast mineralization capacity and bone formation in the RME rat model. After appliance removal, we observed a slight difference in COL-I, Runx2, and ALP levels between the groups. Due to the complexity of cell types in the MPS and signaling networks in osteogenic marker regulation, STAT3 inactivation did not lead to a simultaneous change in COL-I, Runx2, and ALP expression on a microscopic level. Micro-CT, which provided evidence of MPS bone structure on a macroscopic level, showed that the bone volume in the same sized ROI decreased after STAT3 inactivation during expansion. Thus, STAT3 inactivation may delay bone formation in response to mechanical loading. Furthermore, the increased bone volume in the E group after expander displacement indicates continued bone deposition, which was further slowed by STAT3 inactivation.

As a preliminary conclusion from TRAP staining, this study suggests that STAT3 might be involved in osteoclast activity during palatal expansion and post-expansion relapse. STAT3 indirectly mediates osteoclast activity by promoting RANKL expression in osteoblasts to initiate osteoclast differentiation.^37^ In a recent study, STAT3-deficient osteoclast precursors demonstrated reduced osteoclast differentiation.^38^ In our study, STAT3 inhibition disrupted loading-induced bone remodeling by decelerating both bone formation and resorption, leading to a smaller increase in arch width. During post-expansion relapse, bone remodeling continues until a new balance between bone formation and bone resorption is achieved. After 1-week relapse, the E+Stattic group showed greater bone resorption than the E group, indicating STAT3 inhibition might delay the achievement of new bone balance, and predispose MPS to relapse.

However, the study had certain limitations. First, the forces that induce relapse would act for up to several months in clinical practice, and COL-I and Runx2 expressions did not revert to those of the Ctrl group on D14. Therefore, observation of longer relapse periods is required for further investigation. Second, the MPS region has many types of cells, including fibroblasts, osteoblasts, chondrocytes, periosteal cells, and mesenchymal cells. Insights into how these cells are affected by tensile loading *in vitro* will enhance our understanding of the molecular mechanism. Finally, mechanical stimuli initiate bone formation and resorption. In this study, we primarily focused on the former. Future studies will need to delve deeper into the latter to provide a more complete picture.

## Conclusions

Our results suggest that STAT3 inactivation impedes bone formation in the MPS during palatal expansion and post-expansion relapse. STAT3 is mechanosensitive in osteoblasts and can regulate its osteogenic response to mechanical stimuli. Therefore, STAT3 can be developed into a biotherapeutic drug target and requires further study.
